# Efficacy and Tolerability of Repetitive Transcranial Magnetic Stimulation on Suicidal Ideation: A Systemic Review and Meta-Analysis

**DOI:** 10.3389/fpsyt.2022.884390

**Published:** 2022-05-06

**Authors:** Guan-Wei Chen, Tien-Wei Hsu, Pao-Yuan Ching, Chih-Chuan Pan, Po-Han Chou, Che-Sheng Chu

**Affiliations:** ^1^Department of Psychiatry, Kaohsiung Veterans General Hospital, Kaohsiung, Taiwan; ^2^Department of Psychiatry, China Medical University Hsinchu Hospital, China Medical University, Hsinchu, Taiwan; ^3^Center for Geriatric and Gerontology, Kaohsiung Veterans General Hospital, Kaohsiung, Taiwan; ^4^Non-invasive Neuromodulation Consortium for Mental Disorders, Society of Psychophysiology, Taipei, Taiwan; ^5^Graduate Institute of Medicine, College of Medicine, Kaohsiung Medical University, Kaohsiung, Taiwan

**Keywords:** suicidal ideation, repetitive transcranial magnetic stimulation, depression, borderline personality, bipolar disorder

## Abstract

**Objective:**

This study aimed to investigate the efficacy of repetitive transcranial magnetic stimulation (rTMS) in treating suicidal ideation in patients with mental illness.

**Method:**

We followed the Preferred Reporting Items for Systematic Reviews and Meta-Analyses guidelines. Major electronic databases were systematically searched from the time of their inception until July 22, 2021. The primary outcome was the mean change in the scores for suicidal ideation. The secondary outcome was the mean change in depression severity.

**Results:**

Ten randomized controlled trials were eligible with 415 participants in the active treatment group (mean age = 53.78 years; mean proportion of women = 54.5%) and 387 participants in the control group (mean age = 55.52 years; mean proportion of women = 51.78%). rTMS significantly reduced suicidal ideation (k = 10, *n* = 802, Hedges' g = −0.390, 95% confidence interval [CI] = −0.193 to −0.588, *p* <.001) and severity of depressive symptoms (k = 9, *n* = 761, Hedges' g = −0.698, 95% CI = −1.023 to −0.372, *p* < 0.001) in patients with major mental disorders. In the subgroup analysis, rTMS reduced suicidal ideation among patients with non-treatment-resistant depression (non-TRD) (−0.208) but not in those with TRD. rTMS as combination therapy had a larger effect than did monotherapy (−0.500 vs. −0.210). Suicidal ideation significantly reduced in patients receiving more than ten treatment sessions (-0.255). Importantly, the rTMS group showed favorable tolerability without major adverse events.

**Conclusion:**

The study showed that rTMS was effective and well-tolerated in reducing suicidal ideation and depression severity in patients with major mental disorders.

## Introduction

Suicidal behavior is a significant health problem worldwide, accounting for 1.3% of all deaths. More than 700,000 people die by suicide annually. A systematic review of 44 studies from 2000 to 2017 showed that an average of 80% of patients reached out to primary health care in the year prior to suicide (1). Treatments for suicidal patients include psychotherapy, social support intervention, electroconvulsive therapy, and pharmacotherapy using antidepressants, lithium, and clozapine (2). However, owing to the complexity of suicide and associated risk factors, it is difficult to suggest clear treatment guidelines (3).

Mood disorders constitute one-half to two-thirds of all completed suicides (4). A meta-analysis showed that approximately 90% of suicide cases involved a psychiatric disorder, of which approximately 43.2% had some of the affective disorders and 25.7% had issues with substance use (5–7). Among patients with affective disorders, approximately 30%−40% and 50% patients had major depressive disorder (MDD) and bipolar disorder (BD), respectively (8, 9). However, a prospective study showed that BD did not independently influence the risk of suicidal behavior (10). Another study showed that patients with pure major depressive episodes or mixed states in BD had higher risk of suicidal behavior presentation than those with mania, hypomania, and euthymic periods (11). Hence, treatment of depressive episodes in patients with unipolar and bipolar disorder is important for the prevention of suicide attempts.

The effect of psychopharmacology on suicidal outcomes remains unclear because of the heterogeneity of strategies and outcome measures as well as the absence of good standards for evidence level in the literature (2). Another systemic review reported that ketamine and lithium reduced the rate of suicide compared with placebo (12). However, a recent observational study reported that the use of psychotropic medication, including antidepressants and lithium, was not associated with a decrease in suicidal ideation and suicide reattempts (13). Therefore, it is vital to develop more effective and alternative strategies to prevent suicide (2).

Transcranial magnetic stimulation (TMS) is a United States Food and Drug Administration-approved non-invasive brain stimulation technique for treatment-resistant depression (TRD) (14–16). It is also used to treat several psychiatric disorders, such as BD (17), schizophrenia (18), obsessive-compulsive disorder (15, 19), and borderline personality disorder (BPD) (20), all of which led to a higher risk of death from suicide (21). A recent systematic review showed that TMS may be an effective, safe, and well-tolerated technique for treating suicidal behavior, especially in patients with concurrent depression treated with antidepressants (22). Another systematic review of 20 studies, including both randomized controlled trials (RCTs) and open-label trials, found high-frequency (≥ 10 Hz) repetitive TMS over the left dorsolateral pre-frontal cortex to be an adjunct to antidepressants, which significantly reduced suicidal behavior in patients with TRD (23). However, no quantitative outcomes were reported in the meta-analysis method. The results should be cautiously interpreted because of the considerable risk of bias in qualitative studies.

Aside from the above gaps in the literature, no meta-analysis has been performed to estimate the effect of rTMS on suicide-related outcomes. Although some evidence has shown that rTMS is effective in reducing psychiatric symptoms in several mental disorders, the efficacy of rTMS in reducing suicidality remains uncertain. This study aimed to demonstrate the efficacy and safety of rTMS in the treatment of suicidal behavior in major mental disorders. We also compared the effect of rTMS in reducing suicide risk among patients with different psychiatric diagnoses.

## Methods

### Database Searches

This meta-analysis was conducted according to the Preferred Reporting Items for Systematic Reviews and Meta-Analyses guidelines (24) (Supplementary Tables S1A,B). PubMed, Medline, Embase, and Cochrane Library databases were systematically searched from the date of their inception until July 22, 2021 (Supplementary Table S2). The search terms included *brain modulation, rTMS, repetitive transcranial magnetic stimulation, TBS, theta burst stimulation, suicide, suicidality, suicide attempt, and suicide ideation*. Medical subject headings, free text terms, and variations were applied, and Boolean operators (OR, AND) were used to combine the searches. The reference lists of the included articles and recent reviews were also searched to identify additional references. This review was registered in the Prospective Register of Systematic Reviews (PROSPERO, CRD42022269282). Ethical approval was not sought for this study, as it included an analysis of secondary data.

### Eligibility Criteria and Study Selection

The following eligibility criteria were applied: (1) peer-reviewed original articles on clinical trials investigating the effects of rTMS treatment for reducing suicidality; (2) RCTs only; and (3) patients with suicidal ideation without restriction to specific psychiatric disorders. We excluded case series, observational studies, open-label trials, conference abstracts, and trials without a placebo arm (Supplementary Table S3). If there were overlapping data in the studies, only the study with complete data was included in the analyses. Two authors (CS Chu and GW Chen) independently assessed the inclusion/exclusion criteria and selected the studies. Any discrepancies in article retrieval were discussed between the two authors. In the absence of consensus between the two reviewers, a third reviewer (TW Hsu) made the final decision.

### Methodological Quality Assessment

The Jadad score (25) and the Cochrane Risk of Bias version 2 (RoB2) (26) tools were used by the two authors (CS Chu and GW Chen) to assess the methodological quality of the included studies independently and in duplicate. The Jadad score included three categories of study quality: randomization, blindness, and withdrawals and dropouts. The Jadad score ranged from 0 (poor quality) to 5 (high quality). In case of discrepancies, another author (TW Hsu) was consulted to obtain a consensus.

### Data Extraction

The two authors (CS Chu and GW Chen) extracted data from the included studies in accordance with a pre-specified data extraction form independently and in duplicate. Any discrepancies were resolved by a third investigator (TW Hsu). The extracted data included basic characteristics of the participants (mean age and percentage of women), stimulation protocol (stimulation site, pulses per session, total sessions, frequency, and power), combined treatment (antidepressant and other usual treatment), and study quality measured by the Jadad scoring system.

### Primary and Secondary Outcomes

We defined the primary outcome as the mean change in the scores of suicidal ideation between baseline and the end of the last rTMS session, which had been recorded using a validated scale, such as the Beck Scale of Suicidal Ideation (27), suicide item of the Hamilton Rating Scale for Depression (17 items or 24 items) (28), Self-rating Idea of Suicide Scale, Columbia Suicide Severity Rating Scale (29), or suicidal behavior item of Clinical Global Impression Scale for BPD (30).

We defined secondary outcome as the response rate of depression, which was defined as more than 50% reduction of the depressive symptom score from baseline to the end of the last rTMS session. We defined secondary outcome as the response rate of depression, which was defined as more than 50% reduction of the depressive symptom score from baseline to the end of the last rTMS session. We chose improvement of depression as secondary outcome because patients with suicidal ideation are highly comorbid with depression. We want to know if the efficacy of rTMS on suicidal ideation is related to patients' depression. Therefore, we further investigated whether the effect of rTMS on suicidal ideation is independent from depression change by exploring the association between the improvement of depressive severity and reduction of suicidal ideation. We extracted data on the levels of depression based on the most used scales in the included studies. The Hamilton depression rating scale (28) is the most frequently used scale to assess depression severity, followed by the Montgomery-Åsberg Depression Rating Scale (31) or Beck Depression Inventory (BDI) (32). The secondary outcome was the response rate, which was defined as more than 50% reduction of the depressive symptom score from baseline to the end of the last rTMS session.

### Meta-Analysis Procedure

Due to the anticipated heterogeneity across studies, a random-effects meta-analysis was conducted (33). We calculated the Hedges' g statistic as the estimate of the within-group effect size and 95% confidence intervals (CI) for changes from pre-treatment to post-treatment and between-group (intervention group vs. control group) effect size for the primary outcome and mean change in depressive symptoms score. When different scales were used between studies, standardized mean differences between treatment groups were calculated for each trial and used to derive the total estimate of treatment effect on the outcomes. The standardized mean differences offer a summary statistic in meta-analysis when the studies assess the same outcome but with different scales (34). We used the standard error or *t*-value to estimate those without a standard deviation. For interpretation of effect sizes, we followed the rule of classifying <0.2 as very small, 0.2–0.5 as small, 0.5–0.8 as moderate, and >0.8 as large. Odds ratios and 95% CIs were calculated for dichotomous data. All meta-analytic procedures were performed using the Comprehensive Meta-Analysis software, version 2 (Biostat, Englewood, NJ). The threshold for statistical significance was set at a two-tailed *P*-value < 0.05.

### Heterogeneity, Publication Bias, Sensitivity Analysis, Meta-Regression Analyses, and Subgroup Analysis

The Cochran's Q test and I^2^ metric were used to assess heterogeneity. Egger's regression test and funnel plot inspection were used to assess publication bias. Meta-regression analyses were conducted with unrestricted maximum likelihood random effects when data on each potential moderator were used in at least five different studies (35). The mean age, percentage of women, and Jadad scores were considered as variables for the meta-regression analyses. We performed sensitivity testing with the one study removal test to investigate potential confounders by any one of the outliers in the included studies (36). A subgroup meta-analysis was performed when at least three sets of data were available. We conducted a subgroup analysis to explore the potential difference when comparison was done based on the characteristics of the participants who may require special attention. We performed subgroup analyses for different diagnoses (TRD vs. non-TRD) and treatment protocol (rTMS monotherapy vs. rTMS combination therapy; <10 sessions vs. ≥ 10 sessions; rTMS vs. intermittent theta-burst stimulation (iTBS); left dorsolateral pre-frontal cortex (DLPFC) vs. not left DLPFC). The definitions of TRD were based on antidepressant trials Stage I (37, 38) or II (39), Thase and Rush staging model (40), and Stage III or IV (41) in the antidepressant treatment history form (42). We defined those receiving rTMS monotherapy as those: (1) not allowed to receive concurrent treatment with antidepressants (43), (2) at least 2 weeks free from using psychotropic agents except for the habitual use of benzodiazepines, if necessary (37), and (3) 2 weeks free from using antidepressant, antipsychotic, and mood stabilizers (38).

## Results

### Studies in the Meta-Analysis

**Figure 1:**
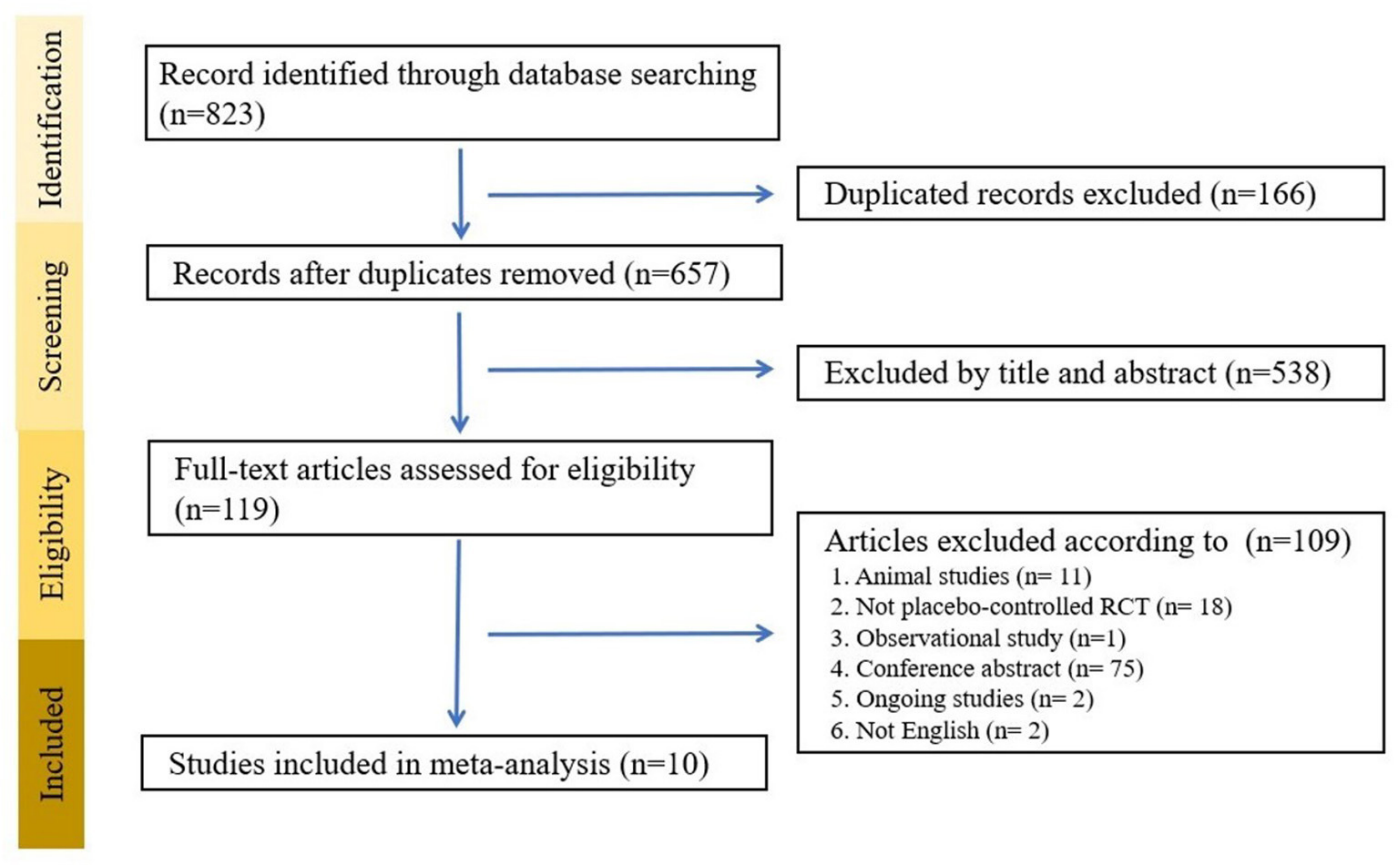
Flow chart of the search strategy.

After searching the database, we identified 823 potential articles, from which we excluded 704 articles after title and abstract screening. We excluded 109 studies through full-text assessment for specific reasons (Supplementary Table S3). Finally, 10 studies satisfied our criteria (Table 1) (37–39, 41, 43–48). A flowchart of the search strategy is presented in Figure 1. A total of 802 participants were included with a mean age of 54.62 (SD = 11.46) years and a mean proportion of women of 53.2% (429/802).

**Table 1:** The characteristics and demographics of the included studies.

**Author (year); Country**	**Population**	**Follow up time**	**Intervention, n Control, n**	**Age (female, %)**	**Stimulation protocol (stimulate site, pulses per session, total sessions, frequency and power)**	**Scales for primary outcome**	**Site targeting**
Desmyter S et al. (38); Belgium	TRD	1 weeks	r-TMS + sham control, 12	44.91 ± 10.8(58.3)	L-DLPFC, 1620 pulses per-session, 20 sessions, 54 triplet bursts within 2s, 100% MT	BSI	Neuro-navigation
George MS et al. (44); USA	Post-traumatic stress disorder	6 months	TAU+ r-TMS, 20 TAU+ sham control, 21	38.7 ± 15(10) 46.1 ± 15.9 (19)	L-DLPFC, 6000 pulses per session, 9 sessions, 10Hz, 120% MT	BSI	N/A
Qin BY et al. (45); China	Elderly patients with depression	4 weeks	Escitalopram + r-TMS, 85 Escitalopram + sham control, 100	70.03 ± 5.97 (67.5) 69.43 ± 5.98 (67.34)	L-DLPFC, 120-2000 pulses per session, 20 sessions, 10Hz, 80%~110% MT	SIOSS	N/A
Yesavage JA et al. (41); USA	TRD	6 months	TAU+ r-TMS, 81 TAU+ sham control, 83	55.6 ± 12.2(33.33) 54.8 ± 12.6(35)	L-DLPFC, 4000 pulses per session, 20-30 sessions, 10Hz, 120% MT	BSI, CSSRS	N/A
Weissman CR et al., (39); Canada	TRD	6 weeks	r-TMS, 128 Sham control, 61	49.26 ± 13.2(61.7) 47.3 ± 12.5(62.3)	L-DLPFC or bil-DLPFC, 1215-2100 pulses per session, 15 sessions, R: 1Hz/ L: 10 Hz, 100-120% MT	Suicide item of HAMD-17	5-cm rule/ structural MRI
Baeken C et al. (37); Belgium	TRD	1 weeks	r-TMS, 21 Sham control, 24	37 ± 18.5(76.2) 47.5 ± 20.75(70.8)	L-DLPFC, 1620 pulses per session, 20 sessions, 54 triplet bursts within 2s, 110% MT	BSI	Neuro-navigation
Rao V et al. (43); USA	MDD after traumatic brain injury	16 weeks	r-TMS, 17 Sham control, 17	39.8 ± 14.2(61.5) 40.2 ± 14.6(35.3)	R-DLPFC, 1200 pulses per session, 20 sessions, 1Hz, 110% MT	BSI	F4 of the International 10–20 System for Electrode Placement
Dai L et al. (46); China	Elderly depression patients	4 weeks	Escitalopram + r-TMS, 62 Escitalopram + sham control, 62	69.99 ± 8.69(63) 67.15 ± 9.9(60)	L-DLPFC, 800 pulses per session, 20 sessions, 10Hz, 100% MT	SIOSS	N/A
Pan F et al. (46); China	MDD	1 weeks	Escitalopram + r-TMS, 21 Escitalopram + sham control, 21	18.14 ± 3.94(90.5) 21.43 ± 6.79(76.2)	L-DLPFC, 6000 pulses per session, 7 sessions, 10Hz, 100% MT	BSI	Neuro-navigation
Calderon-Moctezuma AR et al. (47); Mexico	Borderline personality disorder	3 weeks	TAU+ r-TMS, 9 TAU+ sham control, 9	24 ± 6.29 (71.4) 28.14 ± 8.31 (57.1)	DMPFC, 1500 pulses per session, 15 sessions, 5Hz, 100% MT	Suicidal behavior item in CGI-BPD	N/A

*BSI, Beck scale for suicide ideation; CGI-BPD, Clinical Global Impression Scale for Borderline Personality Disorder; CSSRS, Columbia Suicide Severity Rating Scale; DLPFC, Dorsolateral pre-frontal cortex; DMPFC, dorsomedial pre-frontal cortex; HAMD-17, Hamilton Depression Rating Scale-17; MDD, Major depressive disorder; MT, motor threshold; r-TMS, repetitive transcranial magnetic stimulation; SIOSS, Self-rating Idea of Suicide Scale; TAU, Treatment-As-Usual; TRD, Treatment-resistant depression*.

All 10 studies were RCTs (37–39, 41, 43–48). For the primary and secondary outcomes, available data for further analysis were obtained from 10 studies on the reduction of suicidal ideation (37–39, 41, 43–48). Nine studies included patients with current depressive episodes. The most common diagnosis was MDD in six studies (37–39, 41, 43, 46). One of these included patients had a diagnosis of MDD and traumatic brain injury (TBI) (43). Among the six studies that included MDD cases, four had TRD (37–39, 41). The remaining four studies included cases with BPD (47), depressive disorder (45, 48), and unipolar or bipolar disorder combined with post-traumatic stress disorder or traumatic brain injury (44). The RCTs included 415 participants in the active treatment group (mean age = 53.78 years, SD =11.4; mean proportion of women = 54.5%) and 387 participants in the control group (mean age = 55.52 years, SD =11.5; mean proportion of women = 51.78%) (37–39, 41, 43–48).

### Methodological Quality of the Included Studies

We assessed the quality of the included studies using the Jadad scoring system (25) and the Cochrane Risk of Bias version 2 (RoB2) (26) tools. Across all 10 studies, the average Jadad score was 3 (range: 2–5) (Supplementary Table S4). Five of the 10 studies showed a low overall risk of bias according to RoB2 evaluation. The analysis of the remaining five studies revealed some concerns when one or more domains were judged to be at “some concerns” of bias (Supplementary Table S5). The included studies revealed 50% (5/10) trials rating as “some concerns” of bias mainly arising from measurement of the outcome.

### Handling the Differences in Scales Used to Evaluate the Primary and Secondary Outcome

For the primary outcome, there are five kinds of scales used to evaluate the severity of suicidal ideation. The scales include the Beck Scale for Suicide Ideation, Self-rating Idea of Suicide Scale, Suicidal behavior items of the clinical global impression scale for BPD, Columbia-Suicide Severity Rating Scale, and suicide items in the Hamilton Depression Rating Scale-17. There is no formulation to convert data from one scale to one another. Hence, the standardized mean differences (SMD) between treatment groups were calculated for each trial and used to derive the total estimate of the treatment effect on the outcomes. The SMD is a summary statistic in meta-analysis when the studies assess the same outcome but with different scales (34).” For the secondary outcome, there are four kinds of scales used to evaluate the severity of depression. The scales include the Hamilton Depression Rating Scale-17 (HAMD-17), Hamilton Depression Rating Scale-24 (HAMD-24), BDI-I and BDI-II. We converted BDI-I, BDI-II, and HAMD-24 scores to equivalent HAMD-17 scores based on previous studies (49).

### Primary Outcome: Efficacy of RTMS in Reducing Suicidal Ideation

**Figure 2:**
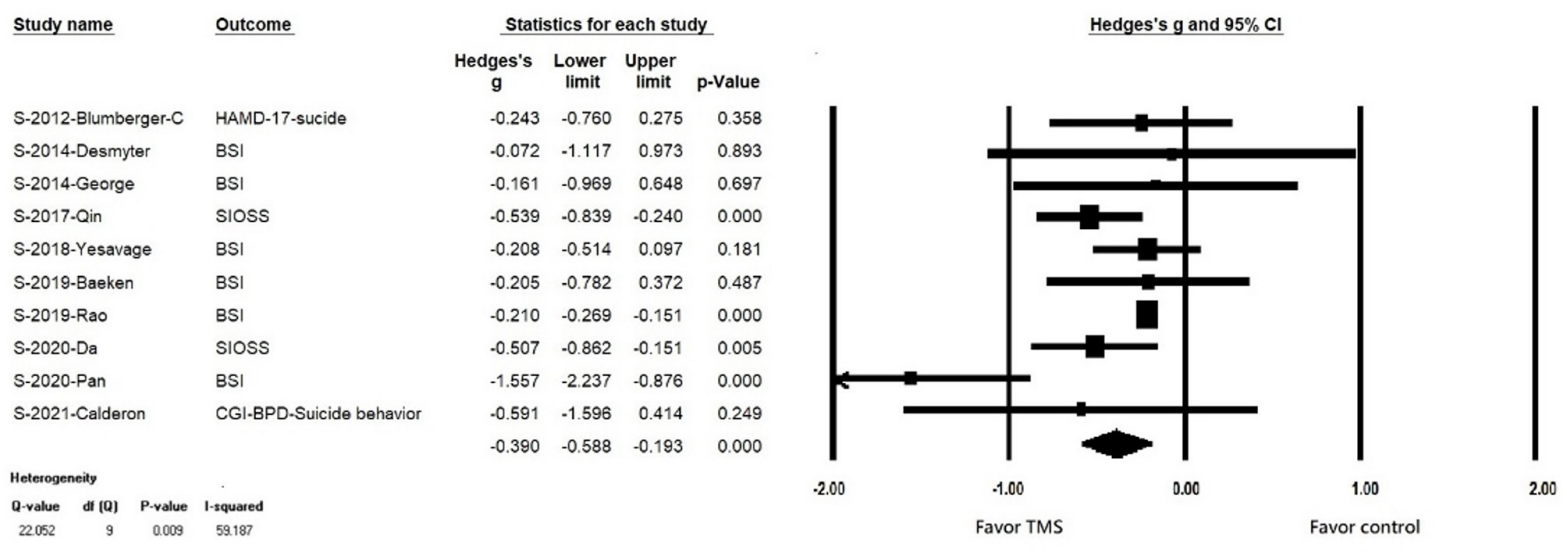
Forest plot of meta-analysis of improvement of suicidal ideation in patients receiving repetitive transcranial magnetic stimulation treatment and in those with control treatment.

In patients with suicidal ideation, rTMS significantly reduced suicidality (k = 10, *n* = 802, Hedges' g = −0.390, 95% CI = −0.193 to −0.588, *p* < 0.001) (Figure 2). There was no evidence of publication bias (Egger's regression test, *p* = 0.117), but significant heterogeneity was observed (Q value = 22.964, I2 = 56.453, *p* = 0.0011). In the sensitivity analysis, the results remained significant, showing the efficacy of rTMS in reducing suicidal ideation after the one study removal test. Furthermore, after removing the study conducted by Pan et al., no significant heterogeneity was found.

### Source of Heterogeneity: Meta-Regression

In the meta-regression analysis, the percentage of females (k=10, slope =-0.994, *p* =0.004) and baseline BSI score (k=6, slope = −0.03136, *p* < 0.016) emerged as significant moderators. Therefore, rTMS was more efficacious in reducing suicidal ideation in the studies with higher percentage of females and higher baseline suicidal severity than those with lower percentage of females and lower baseline suicidal severity. Age, baseline depression severity, treatment duration, improvement of depression severity (change of equivalent HAMD-17 score), and pulses per session did not contribute to heterogeneity (Supplementary Table S6A).

### Source of Heterogeneity: Subgroup Analysis

We conducted five subgroup analyses, including TRD compared with non-TRD, rTMS combination therapy compared with rTMS monotherapy, <10 treatment sessions compared with more than 10 treatment sessions, target site over left DLPFC compared with non-left DLPFC, and rTMS compared with iTBS (Table 2).

**Table 2:** Subgroup analyses of rTMS on suicide ideation reduction and depression symptoms.

	**Improvement in suicide ideation scale (Hedges' g, 95% CI)**	**Improvement in depression scale (Hedges' g, 95% CI)**
**Diagnoses**		
TRD	−0.208 (−0.441 to 0.025) *p* = 0.081, k= 4	– 0.289 (– 0.523 to – 0.055) *p* = 0.015, k= 4
Non-TRD	−0.534 (−0.856 to −0.213) *p* = 0.001, k= 6	−1.054 (−1.432 to −0.677) *p* < 0.001, k= 5
**Treatment**		
rTMS combination therapy^a^	−0.500 (−0.777 to −0.222) *p* < 0.001, k= 7	−0.685 (−0.853 to −0.517) *p* < 0.001, k= 6
rTMS monotherapy	−0.210 (−0.268 to −0.151) *p* < 0.001, k= 3	−0.271 (−0.775 to 0.234) *p* = 0.293, k= 3
**Treatment session**		
<10 sessions	k = 2, not applicable	k = 2, not applicable
10 or more treatment sessions	−0.255 (−0.342 to −0.168) *p* < 0.001, *k* = 8	−0.567 (−0.812 to −0.321) *p* < 0.001, *k* = 8
**Treatment protocol**		
rTMS	−0.427 (−0.651 to −0.202) *p* < 0.001, *k* = 8	−0.799 (−1.179 to −0.419) *p* < 0.001, *k* = 7
iTBS	*k* = 2, not applicable	*k* = 2, not applicable
**Target site**		
Left DLPFC	−0.47 (−0.757 to −0.182) *p* = 0.001, *k* = 7	−0.73 (−1.132 to −0.328) *p* < 0.001, *k* = 6
Not left DLPFC (including Right DLPFC, DMPFC, and bilateral DLPFC)	each *k* = 1, not applicable	each *k* = 1, not applicable

*CI, confidence interval; DLPFC, dorsolateral pre-frontal cortex; DMPFC, dorsomedial prefrontal cortex; Itbs, Intermittent theta burst stimulation; r-TMS, repetitive transcranial magnetic stimulation; TRD, Treatment-resistant depression*.

a *allowed to combine other usual medication or usual treatment*.

**Figure 3:**
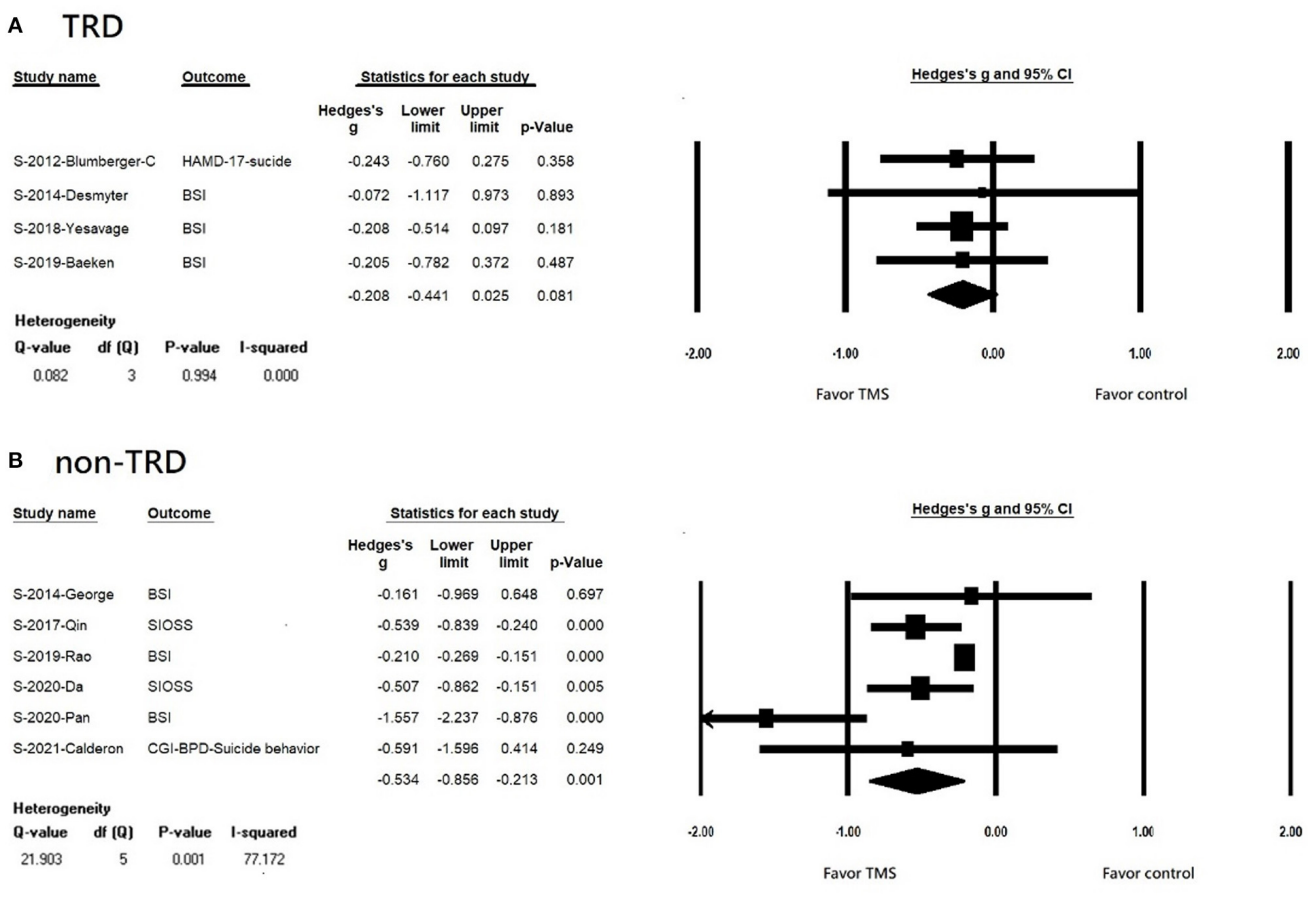
**(A)** Forest plot of meta-analysis of improvement of suicidal ideation in patients with TRD receiving repetitive transcranial magnetic stimulation treatment and in those with control treatment. **(B)** forest plot of meta-analysis of improvement of suicidal ideation in patients with non-TRD receiving repetitive transcranial magnetic stimulation treatment and in those with control treatment.

**Figure 4:**
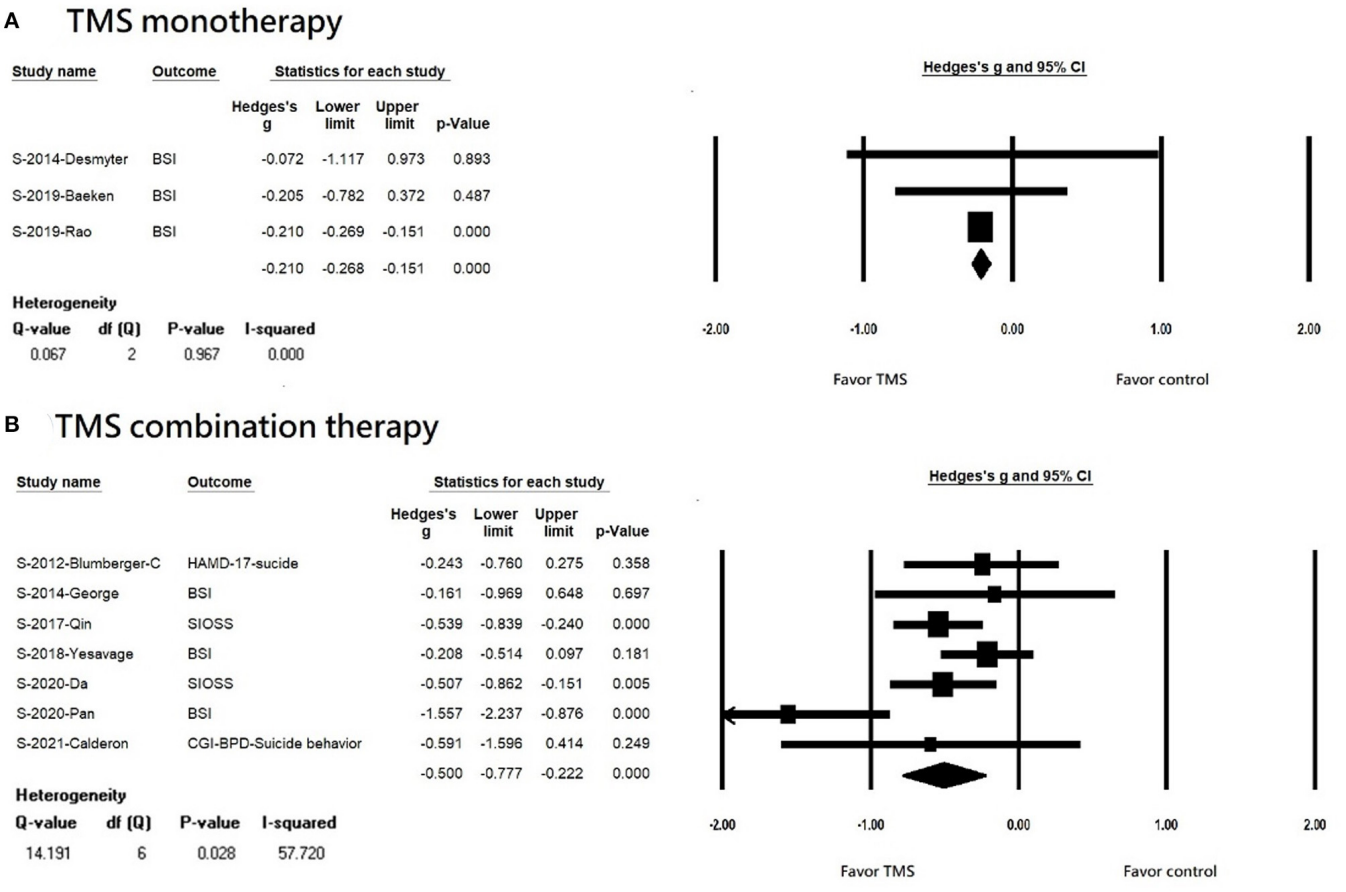
**(A)** Forest plot of meta-analysis of improvement of suicidal ideation in patients receiving repetitive transcranial magnetic stimulation monotherapy and in those with control treatment. **(B)** forest plot of meta-analysis of improvement of suicidal ideation in patients receiving repetitive transcranial magnetic stimulation combination therapy and in those with control treatment.

We found that rTMS reduced suicidal ideation among patients with non-TRD, but not in the TRD population (TRD, k = 4, *n* = 410, Hedges' g = −0.208, 95% CI = −0.441 to 0.025, *p* = 0.081; non-TRD, k = 6, *n* = 444, Hedges' g = −0.534, 95% CI = −0.856 to −0.213, *p* = 0.001) (Figures 3A,B). Both rTMS monotherapy and rTMS combination therapy significantly reduced suicidal ideation (rTMS combined with usual treatment, k = 7, *n* = 715, Hedges' g = −0.500, 95% CI = −0.777 to −0.222, *p* < 0.001; rTMS alone, k = 3, *n* = 87 Hedges' g = −0.210, 95% CI = −0.268 to −0.151, *p* < 0.001) (Figures 4A,B). Patients who received rTMS combined with usual treatment had a significantly greater reduction in suicidal ideation than those who received rTMS monotherapy alone (*p*= 0.005). Patients who underwent more than 10 treatment sessions had a significantly reduced suicidal ideation (10 or more sessions of rTMS, k = 8, *n* = 719, Hedges' g = −0.255, 95% CI = −0.342 to −0.168, *p* < 0.001); however, we could not perform subgroup analysis in those receiving less than 10 treatment sessions because only two studies were available. Patients who received rTMS showed significant reduction in suicidal ideation (k = 8, n = 797, Hedges'g = −0.427, 95% CI = −0.651 to −0.202, *p* < 0.001) (Supplementary Figure S1A); however, we could not perform a subgroup analysis in those receiving iTBS because only two studies were available. Patients who received rTMS over the left DLPFC experienced significantly reduced suicidal ideation (k = 7, *n* = 613, Hedges'g = −0.47, 95% CI = −0.757 to −0.182, *p* = 0.001) (Supplementary Figure S1A). The other three studies targeted the dorsomedial pre-frontal cortex (DMPFC) (47), right DLPFC (43), and bilateral DLPFC (39) respectively. Therefore, we could not perform a subgroup analysis.

Regarding method of targeting, several different kinds of methods were used, including neuro-navigation (37, 38, 46), 5-cm rule (41), mixed 5-cm rule and neuro-navigation (39), 6-cm rule (44), and the International 10–20 System for Electrode Placement (43, 47). However, the remaining two studies (45, 48) did not mention the method of targeting; therefore, subgroup analysis could not be performed.

### Secondary Outcome: The Efficacy of RTMS on Reducing Depressive Symptom Severity

rTMS significantly reduced the severity of depressive symptoms (k = 9, *n* = 761, Hedges' g = −0.697, 95% CI = −1.023 to −0.371, *p* < 0.001). There was no evidence of publication bias (Egger's regression test, t = 0.399, *p* = 0.702), but significant heterogeneity was observed (Q value =24.334, I2 = 67.124, *p* = 0.002). In the sensitivity analysis, the results remained significant, showing the efficacy of rTMS in reducing depressive symptom severity after the one study removal test. Furthermore, after removing the study conducted by Pan et al., no significant heterogeneity was found.

### Source of Heterogeneity of Secondary Outcome: Meta-Regression

In the meta-regression analysis, the percentage of women (k=9, slope = −1.226, *p*= 0.001) and baseline equivalent HAMD-17 score (k=9, slope= −0.109, *p*= 0.001) emerged as significant moderators. Therefore, rTMS was more efficacious in reducing suicidal ideation in the studies with higher percentage of women and higher baseline equivalent HAMD-17 scores than in the studies with lower percentage of women and lower baseline equivalent HAMD-17 scores. Age, treatment duration, and pulses per-session did not explain the heterogeneity (Supplementary Table S6B).

### Source of Heterogeneity of Secondary Outcome: Subgroup Analysis

As shown in Table 2, we found that rTMS reduced depressive severity among patients with both TRD and non-TRD (TRD, k = 4, *n* = 410, Hedges' g = −0.289, 95% CI = −0.523 to −0.055, *p* = 0.015; non-TRD, k = 5, *n* = 403, Hedges' g = −1.054, 95% CI = −1.432 to −0.677, *p* < 0.001). Patients with non-TRD had a significantly greater reduction in depressive severity than those with TRD after rTMS treatment (*p* < 0.001). Patients receiving rTMS combination therapy had a significantly reduced depressive severity, but not for those receiving rTMS monotherapy (rTMS combination therapy, k = 6, *n* = 722, Hedges' g = −0.685, 95% CI = −0.853 to −0.517, *p* < 0.001; rTMS monotherapy, k = 3, *n* = 91; Hedges' g = −0.271, 95% CI = −0.775 to 0.234, *p* = 0.293). Patients who underwent more than 10 treatment sessions had a significantly reduced depressive severity (10 or more sessions of rTMS, k = 8, *n* = 771, Hedges' g = −0.567, 95% CI = −0.812 to −0.321, *p* < 0.001); however, we could not perform subgroup analysis in those receiving <10 treatment sessions since only two studies were available. Patients who received rTMS had a significantly reduced depressive severity (rTMS, k = 7, *n* = 756, Hedges' g = −0.799, 95% CI = −1.179 to −0.419, *p* < 0.001); however, we could not perform a subgroup analysis in those receiving iTBS because only two studies were available. Patients who received rTMS over the left DLPFC experienced significantly reduced depression severity (k = 6, *n* = 572, Hedges'g = −0.73, 95% CI = −1.132 to −0.328, *p* < 0.001). The remaining three studies targeted the DMPFC (47), right DLPFC (43), and bilateral DLPFC (39). Therefore, we could not perform a subgroup analysis.

Regarding method of targeting, several different kinds of methods were used, including neuro-navigation (37, 38, 46), 5-cm rule (41), mixed 5-cm rule and neuro-navigation (39), and the International 10–20 System for Electrode Placement (43, 47). However, the remaining two studies (45, 48) did not mention the method of targeting; therefore, subgroup analysis could not be performed.

### Adverse Effect and Attrition

Most of the included studies reported common adverse effects, such as headaches (39, 41, 43–48) and dizziness (43–45, 47, 48). Other adverse effects such as nausea/vomiting (44, 45, 48), dry mouth (45, 48), eye problems (43, 44), sleep problems (39, 43), constipation (45, 48), and chest tightness (48) have also been reported. The attrition rate ranged from 0% (37, 38) to 55% (44) (Supplementary Table S7).

## Discussion

The main findings of this meta-analysis are as follows: First, rTMS significantly reduced suicidal ideation and improved depressive symptoms in patients with major psychiatric disorders. Second, rTMS significantly reduced suicidal ideation among patients with non-TRD, but not in those with TRD. Third, both rTMS monotherapy and rTMS combination therapy significantly reduced suicidal ideation, and rTMS combination therapy showed significantly better efficacy than rTMS monotherapy. Fourth, rTMS significantly reduced suicidal ideation among patients receiving more than 10 treatment sessions than those receiving <10 sessions. Fifth, meta-regression analysis showed that rTMS demonstrated greater suicidal ideation reduction among women and those with higher baseline depressive severity. Finally, rTMS was well-tolerated, and most adverse events were minor.

### RTMS and Suicidal Ideation

Previous systematic reviews have revealed that rTMS is promising for the reduction of suicide risk (22, 23, 50). The present study found that rTMS reduced both suicidal ideation and depressive symptoms. A previous study demonstrated that a reduction in suicidal risk was mediated by an improvement in depressive severity (51), whereas others did not show this relationship (38). Therefore, it is still unclear whether the impact of rTMS on suicidal ideation reduction was secondary to improvement in depression or mediated by depression. In the present study, meta-regression analysis showed there was no association between the change in the equivalent HAMD-17 score and reduction of suicidal ideation, suggesting the suicidal ideation improvement seems to be independent of depressive severity. However, the number of recruited studies in the present study was relatively small and in most of the studies assessment of suicidal ideation was a secondary outcome measure. More studies are warranted to address this issue.

Regarding meta-regression, we found a significant negative association between outcomes and percentage of women. Studies with a higher percentage of women showed higher likelihood of benefit from rTMS in reducing suicidal ideation. The findings were consistent with that of a previous study that showed an effect of female hormones on the rTMS therapeutic effect. They found that the improvement in the depression score was associated with a higher estradiol/progesterone ratio in premenopausal women (52).

### Subgroup Analysis

The study found that rTMS reduced suicidal ideation among those with non-TRD, but not in those with TRD. Theoretically, patients with TRD tended to have more severe depressive symptoms with expected higher suicidal ideation than those with non-TRD. Among the recruited trials, we found that 60.6% of patients in the TRD group and 12.5% of those in the non-TRD group were stratified as severe depression based on the HAMD (53) or BDI (54) scores; therefore, rTMS may contribute to higher suicidal ideation reduction in those with TRD. However, the present meta-analysis study had contradicting results. Some reasons may explain this inconsistency. First, only four RCTs included patients with TRD. Among these, two studies followed up for only 1 week, which is significantly shorter than that for the non-TRD group (mean follow-up of 9 weeks). A recent meta-analysis and systemic review found that more profound depressive symptom improvement was observed in the follow-up assessments several weeks after accelerated rTMS and intermittent theta burst stimulation, suggesting that clinical improvement has delayed onset after brain stimulation (55). This is consistent with our hypothesis that only 1 week of follow-up after rTMS may not be long enough to detect clinical improvement. Second, more than half of the non-TRD studies (60%) conducted once-daily 10-Hz high frequency (HF)-rTMS stimulation over the left DLPFC over 4–6 weeks; however, half of the studies (50%) used an accelerated protocol with intermittent theta burst stimulation. Given the different profiles and mechanisms of action between stimulation protocols, it may contribute to different efficacies or times to reduce suicidal ideation. Third, 75% of the studies recruited patients with TRD who received rTMS monotherapy, but only 16.7% of the studies recruited non-TRD patients who received rTMS monotherapy. Among the six studies on rTMS combination therapy, three concurrently used escitalopram (45, 46, 48) and another three used combined treatments (41, 44, 47). A previous study has shown that antidepressant treatment is associated with a reduction in suicidal ideation and suicide attempts (56). Therefore, rTMS combination therapy may explain the greater reduction in suicidal ideation than rTMS monotherapy.

Another subgroup analysis found that those who underwent more than 10 treatment sessions had greater suicidal ideation reduction than those who underwent <10 sessions. Although early rTMS studies used as few as 5–10 sessions of treatment, more recent studies have demonstrated that at least 20–30 sessions are needed for better treatment efficacy (57). More number of sessions with high number of pulses per session correlated with better efficacy in the treatment of depression (58, 59). A review summarized the effect of rTMS on neurotransmitters, brain blood flow, brain activity, electrophysiological mechanisms, and functional connectivity, which are related to depression and may also be related to suicidal ideation (60). One study showed that brain-derived neurotrophic factor levels gradually increased with treatment duration. In contrast, inflammatory cytokine levels, such as IL-1b and TNF-a, gradually decreased in patients receiving rTMS treatment (61). Another study found that regional cerebral blood flow significantly increased after 10 sessions of rTMS, but no significant changes were observed during the first rTMS session (62). The evidence indicates that a greater number of sessions are needed to reap the benefit.

### RTMS and Depressive Symptoms

It is well-known that rTMS is an effective treatment for patients with depression by reducing depressive symptom severity (63–65). However, patients without a diagnosis of TRD could also experience depressive symptoms and attempt suicide. The present study focused on patients not only with depressive symptoms, but also specifically focusing on suicidal ideation, which is noteworthy. There is no convincing treatment for suicidal ideation except clozapine for psychosis and lithium for mood disorders (2). A previous study showed that antidepressant treatment seemed to be associated with increased suicidality (66). Therefore, it is important to develop effective treatments for these patients. We found that rTMS had a beneficial effect on depressive symptoms among this group of patients. This result emphasizes that it would be reasonable to consider rTMS as a therapy option in patients with treatment-resistant depressive disorder and suicidal ideation in patients with other psychiatric disorders, such as BPD and unipolar or bipolar spectrum disorder. Previous RCTs showed that rTMS lessened the severity of BPD symptoms (47, 67), and a meta-analysis revealed that rTMS appeared to be effective in the treatment of bipolar depression (68). Our study results are consistent with this finding.

Suicide is a complex multifactorial phenomenon wherein several biological abnormalities, in addition to genetic and environmental factors, may play a role. For example, the decreased protein and mRNA expression of brain-derived neurotrophic factor, dysregulation of the hypothalamic-pituitary-adrenal axis, and neuroimmune functions, particularly for pro-inflammatory cytokines, are involved in the neurobiology of suicide (69). The mechanism by which rTMS reduces suicidal ideation remains unclear. One study showed that rTMS may increase brain-derived neurotrophic factor levels and decrease pro-inflammatory cytokine levels in older patients with refractory depression (61). Furthermore, studies have demonstrated that cortisol levels decrease significantly after using a dexamethasone–corticotrophin-releasing hormone test among subjects after HF-rTMS (70, 71). Taken together, rTMS may reduce suicidal ideation by modulating several different inflammatory pathways, as described above.

### Strength of the Study

There are several strengths of this study. First, although two previous systemic review studies aimed at discussing the role of rTMS in suicidality (22, 23), both involved qualitative synthesis and not a meta-analysis. The present study conducted a meta-analysis, meta-regression, and subgroup analysis to demonstrate the effect of rTMS on suicidality and explore potential sources of heterogeneity across studies. Second, this study has several advantages over the most recent meta-analysis study (72). We included larger sample sizes (802 vs. 566) and a greater number of eligible studies (10 vs. 8) including three additional RCTs (43, 44, 47) and conducted a meta-regression and a subgroup analysis of TRD vs. non-TRD, which was considered as one of the limitations by Cui et al. (72). Third, the present meta-analysis included high-quality RCTs with sham control, providing robust evidence of the efficacy of rTMS in reducing suicidal ideation.

### Limitations

This study has several limitations. First, the present meta-analysis study included relatively few studies with small sample sizes, which may be underpowered to detect statistical difference. Second, according to the RoB2 analysis, 50% of the studies showed concerns of bias. Thus, caution should be exercised when generalizing the results. Third, the protocol of rTMS was different in different study, including the frequency, total pulses per session, power, sessions per day, etc. Variations in the treatment protocol may also have influenced the results. Hence, we conducted a subgroup analysis and meta-regression analysis to minimize this impact. Unfortunately, not all extracted data could be used to conduct a subgroup analysis. For stimulation site, seven out of the ten studies targeted the L-DLPFC. The other three studies targeted the DMPFC, R-DLPFC, and bilateral DLPFC. Therefore, only the effect of rTMS on reducing suicidal ideation in the target site of L-DLPFC could be analyzed. Fourth, three out of the 10 studies were assigned to rTMS monotherapy group due to restriction of concurrent psychotropic medication use. However, the details of the medication usage were not available. Only one study mentioned the details of how the medication washout before randomized was done and the medication they continued to use, like benzodiazepines (37). Hence, we could not perform examination for medication influence on the effects of rTMS on suicidal ideation. Fifth, most of the eligible studies in the present study considered suicidal assessment as a secondary outcome measure. Not all studies demonstrating the role of rTMS on depression examined the suicidal outcome. Selection bias might be noted. However, no publication bias was found in the present study. Furthermore, we found that there was no association between the change in the equivalent HAMD-17 score and reduction of suicidal ideation via meta-regression, suggesting the possible effect of rTMS on suicidality irrespective of depression severity. Finally, the variable assessment scales used for suicidal ideation and depression across the included studies may limit the comparability and synthesis of studies included in this meta-analysis.

## Conclusion

The current meta-analysis of 10 studies involving a total of 802 participants with suicidal ideation found that rTMS was effective in reducing suicidal ideation and depression severity. It was well tolerated, and most adverse events were minor. rTMS combined with other therapies may be more effective than monotherapy. Due to the relatively small sample sizes included in the present study, future studies involving a greater number of participants would help in investigating more covariates and conduct further subgroup analysis to find which stimulation protocol or patient group was more effective in suicide reduction.

## Data Availability Statement

The original contributions presented in the study are included in the article/Supplementary Material, further inquiries can be directed to the corresponding authors.

## Author Contributions

G-WC prepared the manuscript. T-WH and C-SC conceived and designed the study. T-WH, P-YC, and C-CP critically read the manuscript and made important suggestions. P-HC and C-SC, the corresponding authors, take all the responsibility of collecting all the information from the other authors, including the revision of the manuscript and its submission. All authors reviewed the manuscript and had full access to all study data.

## Funding

The study was supported by grant from Kaohsiung Veterans General Hospital (KGVGH-110-051, VGHKS-109-070) and Ministry of Science and Technology, Taiwan (MOST-109-2314-B-075B-001-MY2). The funding source had no role in any process of our study.

## Conflict of Interest

The authors declare that the research was conducted in the absence of any commercial or financial relationships that could be construed as a potential conflict of interest.

## Publisher's Note

All claims expressed in this article are solely those of the authors and do not necessarily represent those of their affiliated organizations, or those of the publisher, the editors and the reviewers. Any product that may be evaluated in this article, or claim that may be made by its manufacturer, is not guaranteed or endorsed by the publisher.
