# Supplementary material for: Efficacy and Tolerability of Repetitive Transcranial Magnetic Stimulation on Suicidal Ideation: A Systemic Review and Meta-Analysis
Source: Front Psychiatry. 2022 May 6;13:884390. doi: 10.3389/fpsyt.2022.884390 (PMC9120615; doi:10.3389/fpsyt.2022.884390)
Supplement: Supplementary file 1 [file Data_Sheet_1.docx]

**Supplementary Table S1A:** PRISMA 2020 flow diagram for new systematic reviews which included searches of databases and registers only

**Identification of studies via databases and registers**

Records identified from

Pubmed (n = 257)

MEDLINE (n = 31)

Embase (n = 393)

Cochrane library (n = 142)

Databases (n = 823)

Records removed *before screening*: Duplicate records removed (n = 166)

**Identification**

Records screened

(n =657)

Records excluded title and abstract (n = 538)

**Screening**

Reports sought for retrieval

(n = 119)

Reports not retrieved

(n = 0)

Reports excluded:

Conference abstract (n = 75)

Duplicated database (n = 0)

Comment (n = 0)

Study protocol (n = 2)

No outcome of interest
(n = 30)

Not English nor chinese (n = 2)

Reports assessed for eligibility

(n = 119)

Studies included in review

(n = 10)

Reports of included studies

(n = 10)

**Included**

**Supplementary Table S1B:** PRISMA 2020 checklist

| **Section and Topic** | **Item #** | **Checklist item** | **Location where item is reported** |
| --- | --- | --- | --- |
| **TITLE** | | |  |
| Title | 1 | Identify the report as a systematic review. | Page 1, title |
| **ABSTRACT** | | |  |
| Abstract | 2 | See the PRISMA 2020 for Abstracts checklist. | Page 1-2, abstract |
| **INTRODUCTION** | | |  |
| Rationale | 3 | Describe the rationale for the review in the context of existing knowledge. | Page 2-3, line 43-78 |
| Objectives | 4 | Provide an explicit statement of the objective(s) or question(s) the review addresses. | Page 3, line 79-84 |
| **METHODS** | | |  |
| Eligibility criteria | 5 | Specify the inclusion and exclusion criteria for the review and how studies were grouped for the syntheses. | Page 3, section 2.2 |
| Information sources | 6 | Specify all databases, registers, websites, organisations, reference lists and other sources searched or consulted to identify studies. Specify the date when each source was last searched or consulted. | Page 3, section 2.1 |
| Search strategy | 7 | Present the full search strategies for all databases, registers and websites, including any filters and limits used. | Page 3, section 2.1 |
| Selection process | 8 | Specify the methods used to decide whether a study met the inclusion criteria of the review, including how many reviewers screened each record and each report retrieved, whether they worked independently, and if applicable, details of automation tools used in the process. | Page 3, section 2.2 |
| Data collection process | 9 | Specify the methods used to collect data from reports, including how many reviewers collected data from each report, whether they worked independently, any processes for obtaining or confirming data from study investigators, and if applicable, details of automation tools used in the process. | Page 4, section 2.4 |
| Data items | 10a | List and define all outcomes for which data were sought. Specify whether all results that were compatible with each outcome domain in each study were sought (e.g. for all measures, time points, analyses), and if not, the methods used to decide which results to collect. | Page 4, section 2.5 |
|  | 10b | List and define all other variables for which data were sought (e.g. participant and intervention characteristics, funding sources). Describe any assumptions made about any missing or unclear information. | Page 3 and 4, section 2.2 and 2.4 |
| Study risk of bias assessment | 11 | Specify the methods used to assess risk of bias in the included studies, including details of the tool(s) used, how many reviewers assessed each study and whether they worked independently, and if applicable, details of automation tools used in the process. | Page 3, section 2.3 |
| Effect measures | 12 | Specify for each outcome the effect measure(s) (e.g. risk ratio, mean difference) used in the synthesis or presentation of results. | Page 4, section 2.6 |
| Synthesis methods | 13a | Describe the processes used to decide which studies were eligible for each synthesis (e.g. tabulating the study intervention characteristics and comparing against the planned groups for each synthesis (item #5)). | Page 3, section 2.2 |
|  | 13b | Describe any methods required to prepare the data for presentation or synthesis, such as handling of missing summary statistics, or data conversions. | Page 4, section 2.4 |
|  | 13c | Describe any methods used to tabulate or visually display results of individual studies and syntheses. | Page 4, section 2.6 |
|  | 13d | Describe any methods used to synthesize results and provide a rationale for the choice(s). If meta-analysis was performed, describe the model(s), method(s) to identify the presence and extent of statistical heterogeneity, and software package(s) used. | Page 4, section 2.7 |
|  | 13e | Describe any methods used to explore possible causes of heterogeneity among study results (e.g. subgroup analysis, meta-regression). | Page 4, section 2.7 |
|  | 13f | Describe any sensitivity analyses conducted to assess robustness of the synthesized results. | Page 4, section 2.7 |
| Reporting bias assessment | 14 | Describe any methods used to assess risk of bias due to missing results in a synthesis (arising from reporting biases). | Page 3, section 2.3 |
| Certainty assessment | 15 | Describe any methods used to assess certainty (or confidence) in the body of evidence for an outcome. | Page 4, section 2.6 |
| **RESULTS** | | |  |
| Study selection | 16a | Describe the results of the search and selection process, from the number of records identified in the search to the number of studies included in the review, ideally using a flow diagram. | Page 5, section 3.1, Figure 1 |
|  | 16b | Cite studies that might appear to meet the inclusion criteria, but which were excluded, and explain why they were excluded. | Page 5, section 3.1, Supplementary Table S3 |
| Study characteristics | 17 | Cite each included study and present its characteristics. | Page 5, section 3.1, Table 1 |
| Risk of bias in studies | 18 | Present assessments of risk of bias for each included study. | Page 5, section 3.2, Supplementary Table S4 and S5 |
| Results of individual studies | 19 | For all outcomes, present, for each study: (a) summary statistics for each group (where appropriate) and (b) an effect estimate and its precision (e.g. confidence/credible interval), ideally using structured tables or plots. | Page 6, Section 3.4, 3.7 |
| Results of syntheses | 20a | For each synthesis, briefly summarise the characteristics and risk of bias among contributing studies. | Page 5, section 3.2 |
|  | 20b | Present results of all statistical syntheses conducted. If meta-analysis was done, present for each the summary estimate and its precision (e.g. confidence/credible interval) and measures of statistical heterogeneity. If comparing groups, describe the direction of the effect. | Page 6, Section 3.5, 3.6, 3.8, 3.9 |
|  | 20c | Present results of all investigations of possible causes of heterogeneity among study results. | Page 6, Section 3.5, 3.6, 3.8, 3.9 |
|  | 20d | Present results of all sensitivity analyses conducted to assess the robustness of the synthesized results. | Page 6, Section 3.6, 3.9 |
| Reporting biases | 21 | Present assessments of risk of bias due to missing results (arising from reporting biases) for each synthesis assessed. | Page 5, section 3.2, Supplementary Table S4 and S5 |
| Certainty of evidence | 22 | Present assessments of certainty (or confidence) in the body of evidence for each outcome assessed. | Page 6, Section 3.4, 3.7 |
| **DISCUSSION** | | |  |
| Discussion | 23a | Provide a general interpretation of the results in the context of other evidence. | Page 8, section 4 |
|  | 23b | Discuss any limitations of the evidence included in the review. | Page 10, section 4.5 |
|  | 23c | Discuss any limitations of the review processes used. | Page 10, section 4.5 |
|  | 23d | Discuss implications of the results for practice, policy, and future research. | Page 11, section 5 |
| **OTHER INFORMATION** | | |  |
| Registration and protocol | 24a | Provide registration information for the review, including register name and registration number, or state that the review was not registered. | Page 3, section 2.1 |
|  | 24b | Indicate where the review protocol can be accessed, or state that a protocol was not prepared. | Page 3, section 2.1 |
|  | 24c | Describe and explain any amendments to information provided at registration or in the protocol. | Page 10, section 4.5, third point of limitation |
| Support | 25 | Describe sources of financial or non-financial support for the review, and the role of the funders or sponsors in the review. | Page 11, section 8 |
| Competing interests | 26 | Declare any competing interests of review authors. | Page 11, section 6 |
| Availability of data, code and other materials | 27 | Report which of the following are publicly available and where they can be found: template data collection forms; data extracted from included studies; data used for all analyses; analytic code; any other materials used in the review. | Page 15, section 11 |

*From:*  Page MJ, McKenzie JE, Bossuyt PM, Boutron I, Hoffmann TC, Mulrow CD, et al. The PRISMA 2020 statement: an updated guideline for reporting systematic reviews. BMJ 2021;372:n71. doi: 10.1136/bmj.n71

For more information, visit: <http://www.prisma-statement.org/>

**Supplementary Table S2:** Database searches

Date through 20210722

Total: 658 after removing duplicated

Pubmed: 257

(Brain modulation or rTMS or repetitive transcranial magnetic stimulation or TBS or theta burst stimulation) AND (suicide or suicidality or suicide attempt or suicide ideation) with limitation of human species

Medline: 31

(Brain modulation or rTMS or repetitive transcranial magnetic stimulation or TBS or theta burst stimulation) AND (suicide or suicidality or suicide attempt or suicide ideation

Embase: 393

(Brain modulation or rTMS or repetitive transcranial magnetic stimulation or TBS or theta burst stimulation) AND (suicide or suicidality or suicide attempt or suicide ideation)

Cochrane clinical trial: 142

(Brain modulation or rTMS or repetitive transcranial magnetic stimulation or TBS or theta burst stimulation) AND (suicide or suicidality or suicide attempt or suicide ideation)

**Supplementary Table S3:** Excluded references with reasons

**Animal studies (1-11) n=11**

1. Gao, C., et al. (2019). "Antidepressive effects of taraxacum officinale in a mouse model of depression are due to inhibition of corticosterone levels and modulation of mitogen-activated protein kinase phosphatase-1 (Mkp-1) and brain-derived neurotrophic factor (Bdnf) expression." Medical Science Monitor 25: 389-394.

2. Martin, C. B., et al. (2013). "RNA splicing and editing modulation of 5-HT(2C) receptor function: relevance to anxiety and aggression in VGV mice." Mol Psychiatry 18(6): 656-665.

3. Merson, T., et al. (2017). "Ablation of NG2 glia in the CNS induces anxiety-like behaviour in adult mice." Journal of Neurochemistry 142: 82.

4. Mohammadi-Farani, A., et al. (2021). "Intra-prefrontal cyclosporine potentiates ketamine-induced fear extinction in rats." Exp Brain Res.

5. Ookuma, K., et al. (1990). "Evidence for feeding elicited through antihistaminergic effects of tricyclic antidepressants in the rat hypothalamus." Psychopharmacology (Berl) 101(4): 481-485.

6. Ookuma, K., et al. (1993). "Neuronal histamine in the hypothalamus suppresses food intake in rats." Brain Research 628(1-2): 235-254.

7. Santangelo, A., et al. (2016). "Brain histamine depletion enhances motor sequences complexity of mice tested in open field: New insights from temporal pattern analysis." European Neuropsychopharmacology 26: S279.

8. Seewoo, B., et al. (2019). "Validation of the chronic restraint stress model of depression in rats and investigation of standard vs accelerated rTMS treatment." Neuropsychopharmacology 44: 122-123.

9. Wróbel, A., et al. (2020). "O-1602, an Agonist of Atypical Cannabinoid Receptors GPR55, Reverses the Symptoms of Depression and Detrusor Overactivity in Rats Subjected to Corticosterone Treatment." Frontiers in Pharmacology 11.

10. Yang, Y., et al. "Proteomics reveals energy and glutathione metabolic dysregulation in the prefrontal cortex of a rat model of depression." Neuroscience 247: 191-200.

11. Yoshimatsu, H., et al. (1993). "Ginsenoside Rg1 prevents histaminergic modulation of rat adaptive behavior from elevation of ambient temperature." Physiology and Behavior 53(1): 1-4.

**Not placebo-controlled RCT(12-29) n=18**

1. Barredo, J., et al. (2021). "Multimodal Elements of Suicidality Reduction After Transcranial Magnetic Stimulation." Neuromodulation.
2. Berlim, M. T., et al. (2014). "Augmenting antidepressants with deep transcranial magnetic stimulation (DTMS) in treatment-resistant major depression." World J Biol Psychiatry 15(7): 570-578.
3. Bloch, Y., et al. (2008). "Repetitive transcranial magnetic stimulation in the treatment of depression in adolescents: an open-label study." J ect 24(2): 156-159.
4. Croarkin, P. E., et al. (2018). "High-frequency repetitive TMS for suicidal ideation in adolescents with depression." J Affect Disord 239: 282-290.
5. Davila, M. C., et al. (2019). "Repetitive transcranial magnetic stimulation (rTMS) using different TMS instruments for major depressive disorder at a suburban tertiary clinic." Mental Illness 11(1).
6. Garcia, K. S., et al. (2010). "Repetitive transcranial magnetic stimulation treats postpartum depression." Brain Stimul 3(1): 36-41.
7. Hadley, D., et al. (2011). "Safety, tolerability, and effectiveness of high doses of adjunctive daily left prefrontal repetitive transcranial magnetic stimulation for treatment-resistant depression in a clinical setting." J ect 27(1): 18-25.
8. Holtzheimer, P. E., 3rd, et al. (2010). "Accelerated repetitive transcranial magnetic stimulation for treatment-resistant depression." Depress Anxiety 27(10): 960-963.
9. Keshtkar, M., et al. (2011). "Repetitive transcranial magnetic stimulation versus electroconvulsive therapy for the treatment of major depressive disorder, a randomized controlled clinical trial." J ect 27(4): 310-314.
10. Lewis, C. P., et al. (2019). "Preliminary evidence of an association between increased cortical inhibition and reduced suicidal ideation in adolescents treated for major depression." J Affect Disord 244: 21-24.
11. Lewis, C. P., et al. (2018). "Cortical inhibitory markers of lifetime suicidal behavior in depressed adolescents." Neuropsychopharmacology 43(9): 1822-1831.
12. Pallanti, S., et al. (2014). "rTMS in resistant mixed states: an exploratory study." J Affect Disord 157: 66-71.
13. Stivala, A., et al. (2020). "An open-label feasibility trial of repetitive transcranial magnetic stimulation (rTMS) in depressed pacific islander adolescents." Indian Journal of Psychiatry 62(7): S160.
14. Wall, C. A., et al. (2016). "Magnetic Resonance Imaging-Guided, Open-Label, High-Frequency Repetitive Transcranial Magnetic Stimulation for Adolescents with Major Depressive Disorder." Journal of Child and Adolescent Psychopharmacology 26(7): 582-589.
15. Wall, C. A., et al. (2011). "Adjunctive use of repetitive transcranial magnetic stimulation in depressed adolescents: a prospective, open pilot study." J Clin Psychiatry 72(9): 1263-1269.
16. Zhang, T., et al. (2021). "An Open-label Trial of Adjuvant High-frequency Left Prefrontal Repetitive Transcranial Magnetic Stimulation for Treating Suicidal Ideation in Adolescents and Adults with Depression." Journal of ECT 37(2): 140-146.
17. Zhang, T., et al. (2021). "An open-label trial of adjuvant high-frequency left prefrontal repetitive transcranial magnetic stimulation for treating suicidal ideation in adolescents and adults with depression." The Journal of ECT, 37(2), 140-146.
18. Zhu, J. J., et al. (2019). "Effect of accelerated repetitive transcranial magnetic stimulation on suicide ideation in depressive patients." Journal of Shanghai Jiaotong University (Medical Science) 39(5): 534-538.

**Observational studies (30) n= 1**

1. Abdelnaim, M. A., et al. (2020). "Anti-Suicidal Efficacy of Repetitive Transcranial Magnetic Stimulation in Depressive Patients: A Retrospective Analysis of a Large Sample." Frontiers in Psychiatry 10.

**Conference abstracts (31-105) n= 75**

1. Aiken, E., et al. (2020). "One-Year Clinical Outcomes Following Theta Burst Stimulation for Posttraumatic Stress Disorder." Biol Psychiatry 87(9): S128.
2. Arns, M., et al. (2019). "New insights into precision medicine and target engagement in neuromodulation depression treatments." Encephale 45: S58.
3. Baeken, C. (2017). "Brain influences of accelerated rTMS in major depression." Brain Stimul 10(2): 365.
4. Baeken, C. (2019). "Accelerated intermittent theta burst stimulation rapidly attenuates suicide ideation in major depression: insights from brain perfusion and functional connectivity." Brain Stimul 12(2): 464.
5. Baeken, C. (2019). "Accelerated intermittent theta burst stimulation rapidly attenuates suicide ideation in major depression: insights from brain perfusion and functional connectivity." Brain Stimul 12(2): 464.
6. Baeken, C. (2019). "Accelerated neurostimulation in major depression: insights from brain imaging." Brain Stimul 12(2): 464.
7. Baeken, C., et al. (2018). "Accelerated intermittent theta burst stimulation rapidly attenuates depressive symptoms and suicide ideation in major depression: Insights from brain perfusion and functional connectivity." Biol Psychiatry 83(9): S54.
8. Barredo, J., et al. (2021). "A Solid Foundation: Structure-Function Relationships and Neuromodulation for Posttraumatic Stress Disorder and Suicide." Biol Psychiatry 89(9): S38-S39.
9. Barredo, J., et al. (2019). "Neuroimaging of transcranial magnetic stimulation for suicidality." Neuropsychopharmacology 44: 263-264.
10. Bentzley, B., et al. (2019). "Accelerated intermittent theta-burst stimulation suppresses suicidal ideation in patients with treatment-resistant depression." Brain Stimul 12(2): 400-401.
11. Blavignac, J., et al. (2019). "P.749 Combination extended-release Naltrexone/Bupropion causes significant weight loss without worsening psychiatric symptoms: pooled analysis from the contrave obesity research program." European Neuropsychopharmacology 29: S503-S504.
12. Blavignac, J., et al. (2019). "P.749 Combination extended-release Naltrexone/Bupropion causes significant weight loss without worsening psychiatric symptoms: pooled analysis from the contrave obesity research program." European Neuropsychopharmacology 29: S503-S504.
13. Bloch, Y., et al. (2015). "rTMS in the treatment of adolescent depression." Brain Stimul 8(2): 341.
14. Bodén, R., et al. (2019). "Treatment resistant depression with partial effect of electroconvulsive treatment achieving long lasting remission with dorsomedial prefrontal intermittent theta-burst stimulation – a case report." Brain Stimul 12(2): 499-500.
15. Cailhol, L., et al. (2009). "Transcranial magnetic stimulation for borderline personality disorder: Rationale, stimulation site and methods." European Psychiatry 24: S768.
16. Calati, R., et al. (2008). "Serotonin transporter: An example of gene influence on human behaviour." European Neuropsychopharmacology 18(S4): S205-S206.
17. Chattopadhyay, A., et al. (2019). "Intermittent theta burst stimulation (iTBS) for managing treatment resistant depression." Indian Journal of Psychiatry 61(9): S624.
18. Chin-Lun Hung, G. (2019). "Proceedings #3: Effects of Combining Transcranial Direct Current Stimulation with Mindfulness Training in Patients with Treatment-Resistant Depression: A Pilot Study." Brain Stimul 12(2): e59-e60.
19. Cole, E., et al. (2019). "Accelerated intermittent theta-burst stimulation for treatment-resistant depression in patients with alcohol-use disorder." Brain Stimul 12(2): 505-506.
20. Cole, E., et al. (2020). "Novel non-invasive brain stimulation protocol to rapidly ensure the safety of inpatients with depression & suicidality." Clinical Neurophysiology 131(4): e21.
21. Courtet, P. (2012). "Cognitive and emotional endophenotypes of suicidal behaviour: Are they useful?" European Psychiatry 27.
22. Croarkin, P. (2019). "Recent developments in non-invasive brain stimulation for adolescents with major depressive disorder." Brain Stimul 12(2): 411.
23. Croarkin, P., et al. (2017). "Reduced suicidality and enhanced neuronal integrity with high frequency repetitive transcranial magnetic stimulation treatment in adolescents." Neuropsychopharmacology 43: S355-S356.
24. Croarkin, P., et al. (2019). "Monitoring and modulating adolescent depression and suicidality." Brain Stimul 12(2): 394.
25. Croarkin, P. E. (2018). "Recent Transcranial Magnetic Stimulation Biomarker and Intervention Studies for Adolescent Suicidality." Journal of the American Academy of Child and Adolescent Psychiatry 57(10): S317.
26. Cullen, K. R. and D. A. Brent (2016). "Treatment-resistant depression in adolescents: Neurobiology and novel approaches for treatment." Journal of the American Academy of Child and Adolescent Psychiatry 55(10): S283.
27. Cullen, K. R. and G. J. Emslie (2018). "Applications of Transcranial Magnetic Stimulation to Understand and Treat Adolescents With Depressive Disorders and ASD." Journal of the American Academy of Child and Adolescent Psychiatry 57(10): S317.
28. Daskalakis, J. (2019). "Therapy-oriented induction of seizures." Brain Stimul 12(2): 538.
29. Daskalakis, Z. (2019). "Neurophysiological Mechanisms of rTMS Efficacy in Treatment Resistant Depression." Brain Stimul 12(2): 544.
30. Dolgoff, R., et al. (2013). "Repetitive transcranial magnetic stimulation as a treatment for depression: Outcomes and adverse effects in a community psychiatric practice." CNS Spectrums 18(6): 359.
31. Downar, J., et al. (2018). "Distinctive mechanisms of action for DLPFC-, DMPFC-, and OFC-rTMS in major depression." Biol Psychiatry 83(9): S47-S48.
32. Dwiel, L., et al. (2019). "Decoding impulsive decision-making from rat cortical-striatal oscillations." Neuropsychopharmacology 44: 239.
33. Farzan, F. (2019). "Transcranial magnetic stimulation and electroencephalography predictors of response to rTMS in youth depression." Brain Stimul 12(2): 586.
34. García-Anaya, M. L., et al. (2010). "Differences in the antidepressant effect of right and left repetitive transcranial magnetic stimulation assessed item by item with Hamilton depression rating scale." Bipolar Disord 12: 21.
35. García-Bea, A., et al. (2012). "Increased 5HT2A receptor binding in frontal cortex of schizophrenic subjects: Effect of aging and antipsychotic drug treatment." International Journal of Neuropsychopharmacology 15: 115.
36. George, M. S., et al. (2013). "A two-site pilot study suggests that three days (9 sessions) of high dose left prefrontal repetitive transcranial magnetic stimulation (rTMS) is feasible, safe, and reduces suicidal thinking in suicidal inpatients." Neuropsychopharmacology 38: S421-S422.
37. Goff, D. (2012). "Possibilities and impossibilities of TMS for the treatment of hallucinations." Schizophrenia Research 136: S22.
38. Heeramun, V. and V. Alluri (2016). "Repetitive transcranial magnetic stimulation (rTMS) - A promising treatment for concomitant depression and migraines." Journal of Neuropsychiatry and Clinical Neurosciences 28(3): e47.
39. Hong, E. (2011). "Moderate dose varenicline treatment on neurobiological and cognitive biomarkers in schizophrenia smokers and non-smokers." Neuropsychopharmacology 36: S97-S98.
40. Jensen, E., et al. (2020). "Benchmarking aiTBS With the Gold Standard." Biol Psychiatry 87(9): S129.
41. Johnson, M. D., et al. (2011). "Meeting highlights on psychiatric clinical challenges and advancing the science of treatment: The 3rd annual chair summit." Health Outcomes Research in Medicine 2(3): e169-e182.
42. Kim, J. I. (2015). "Treatment resistant major depression: Initial clinical outcomes and considerations for clinical practice." Brain Stimul 8(2): 344.
43. Kim, K. S. and E. J. Jung (2011). "An open label pilot study of transcranial magnetic stimulation for patients with bipolar depression." European Neuropsychopharmacology 21: S423-S424.
44. Kumar, N. and S. Jha (2013). "Repetitive Transcranial Magnetic Stimulation (RTMS) in organic personality and behavioral disorder, secondary to suicide attempt by hanging." Clinical Neurophysiology 124(10): e165-e166.
45. Lee, J., et al. (2019). "Combination Theta-Burst Stimulation and Cognitive Training for Youth Depression." Biol Psychiatry 85(10): S181‐.
46. Legarreta, M., et al. (2019). "Orbitofrontal functional connectivity and perceived pain disability in veterans with suicide ideation and suicide attempts." Neuropsychopharmacology 44: 292-293.
47. Levine, A., et al. (2016). "The role of social involvement in ameliorating fear, anxiety, and depression: Findings in mice and humans." Journal of the American Academy of Child and Adolescent Psychiatry 55(10): S296.
48. Maslenikov, N., et al. (2010). "Cognitive responce as a predictor of repetitive transcranial magnetic stimulation (rTMS) efficacy in schizophrenia." European Neuropsychopharmacology 20: S448-S449.
49. Mathew, S. J. (2013). "Ketamine and NMDA receptor modulation for treatment-resistant mood disorders." Behavioural pharmacology 24: e8‐e9.
50. Moctezuma, A. C., et al. (2019). "IMPROVEMENT IN BORDERLINE PERSONALITY DISORDER SYMPTOMS WITH DORSOMEDIAL PREFRONTAL CORTEX rTMS: TWO CASES." Brain Stimul 12(2): 522-523.
51. Monteiro, D. C., et al. (2017). "Electroconvulsive therapy followed by repetitive transcranial magnetic stimulation to severe depression with suicide ideation." Journal of ECT 33(3): 215-216.
52. Morilak, D. A., et al. (2009). "Cognitive and emotional plasticity in mood disorders and suicidal behavior." Biol Psychiatry 65(8): 164S.
53. Nemeroff, C. B. (2009). "Neurobiological predictors of treatment response in major depression." International Journal of Psychiatry in Clinical Practice 13: 13.
54. Östlund, H., et al. (2003). Estrogen Receptor Gene Expression in Relation to Neuropsychiatric Disorders. 1007: 54-63.
55. Oviedo, G., et al. (2013). "Trends in the administration of electroconvulsive therapy for schizophrenia in Colombia. Descriptive study and literature review." Eur Arch Psychiatry Clin Neurosci 263(1): S98.
56. Philip, N., et al. (2020). "Transcranial magnetic stimulation in us military veterans-a naturalistic study in the veterans health administration." Neuropsychopharmacology 45: 310-311.
57. Philip, N., et al. (2019). "One-year clinical outcomes following theta burst stimulation for PTSD." Neuropsychopharmacology 44: 267.
58. Phillips, A. L., et al. (2020). "Stanford Accelerated Intelligent Neuromodulation Therapy (SAINT-TRD) induces rapid remission from treatment-resistant depression in a double-blinded, randomized, and controlled trial." Brain Stimul 13(6): 1859‐1860.
59. Pregelj, P. (2011). "Genetics of schizophrenia and bipolar disorder." European Psychiatry 26.
60. Reilmann, R., et al. (2018). "Legato- HD study: a phase 2 study assessing the efficacy and safety of laquinimod as a treatment for Huntington disease." Journal of neurology, neurosurgery and psychiatry 89: A99‐.
61. Seok, J. H. and M. H. Chung (2015). "The efficacy and safety of accelerated repetitive transcranial magnetic stimulation in major depressive disorder: singleblind, randomized study." Brain stimulation. 8(2): 384.
62. Shejekar, S., et al. (2020). "Future of psychiatry." Indian Journal of Psychiatry 62(7): S121.
63. Stimpson, K., et al. (2019). "Rapid Theta Burst Transcranial Magnetic Stimulation in a Hospitalized Patient with Schizophrenia Post-Suicide Attempt is Both Safe and Effective." Brain Stimul 12(2): 408.
64. Sun, Y., et al. (2017). "Neuromodulation of subgenual cingulate activity localizable from EEG." BMC Neuroscience 18.
65. Szyf, M. (2012). "Epigenetic dysregulations in psychoaffective disorders." European Neuropsychopharmacology 22: S124.
66. Tolliver, B., et al. (2016). "Comparison of the implicit association test with established clinical rating scales in suicide risk assessment: baseline data from the better resiliency among veterans and non-veterans with omega-3 s (BRAVO) study." Neuropsychopharmacology 41: S487‐S488.
67. Trejo Cruz, G., et al. (2019). "Clinical effectiveness of 5Hz Transcranial Magnetic Stimulation applied on Left Dorsolateral Prefrontal Cortex and Dorsomedial Prefrontal Cortex on Clinical Depressed Patients." Brain Stimul 12(2): 527.
68. Vismara, M. E. M., et al. (2019). "P.539 Augmentative repetitive TMS in the treatment of poor responder depressed patients: a follow up study." European Neuropsychopharmacology 29: S380-S381.
69. Voineskos, D., et al. (2019). "Is Depression an Illness of Cortical Activation?" Biol Psychiatry 85(10): S340.
70. Voineskos, D., et al. (2017). "Cortical inhibition as a high potential biomarker of response across brain stimulation modalities in treatment resistant depression." Biol Psychiatry 81(10): S29.
71. Weissman, C., et al. (2017). "Bilateral repetitive transcranial magnetic stimulation (RTMS) decreases suicidality in adults with treatment resistant depression." Biol Psychiatry 81(10): S331.
72. Williams, N. (2019). "Accelerated Intermittent Theta Burst Stimulation for Acute Suicidality in an Inpatient Setting." Brain Stimul 12(2): 538-539.
73. Williams, N. (2019). "Stanford Accelerated Intelligent Neuromodulation Therapy for Suicidal Ideation (SAINT-SI)." Biol Psychiatry 85(10): S28‐.
74. Wout-Frank, M. V., et al. (2020). "Exploring the One-Year Clinical Outcomes Following Theta Burst Stimulation for Posttraumatic Stress Disorder." Biol Psychiatry 87(9): S78.
75. Yesavage, J. (2013). "Design and initiation of a department of veterans' Affairs Cooperative Study on the use of repetative Transcranial Magnetic Stimulation (rTMS) in treatment resistant depression." Clinical Neurophysiology 124(10): e121.

**Ongoing studies without available data (105-107) n=2**

1. Bozzay, M. L., et al. (2020). "Combined transcranial magnetic stimulation and brief cognitive behavioral therapy for suicide: study protocol for a randomized controlled trial in veterans." Trials 21(1): 924.
2. Wang, H., et al. (2013). "Efficacy of repetitive transcranial magnetic stimulation in the prevention of relapse of depression: study protocol for a randomized controlled trial." Trials 14: 338.

**Not english nor chinese (107-109) n=2**

1. Bulteau, S., et al. (2019). "[Mood disorders: When should we use repetitive transcranial magnetic stimulation?]." Presse Med 48(6): 625-646.
2. Leong, K., et al. (2020). "A Randomized Sham-controlled Trial of 1-Hz and 10-Hz Repetitive Transcranial Magnetic Stimulation (rTMS) of the Right Dorsolateral Prefrontal Cortex in Civilian Post-traumatic Stress Disorder." Canadian Journal of Psychiatry.

| First Author (year) | Randomization | Blinding | Withdrawl and drop out | | Total scores |
| --- | --- | --- | --- | --- | --- |
| Calderon-Moctezuma AR, 20211 | 1 | 0 | 1 | 2 | |
| Qin BY, 2017 | 1 | 0 | 1 | 2 | |
| George MS, 2014 | 2 | 2 | 1 | 5 | |
| Yesavage JA, 2018 | 2 | 2 | 1 | 5 | |
| Dai L, 2020 | 1 | 1 | 1 | 3 | |
| Pan F, 2020 | 2 | 1 | 1 | 4 | |
| Rao V, 2019 | 1 | 0 | 1 | 2 | |
| Baeken C, 2019 | 1 | 1 | 1 | 3 | |
| Desmyter S, 2014 | 1 | 0 | 1 | 2 | |
| Weissman CR, 2018, | 1 | 1 | 0 | 2 | |

**Supplementary Table S4:** Jadad Quality Scale scores of included studies

**Supplementary Table S5:** Risk of bias


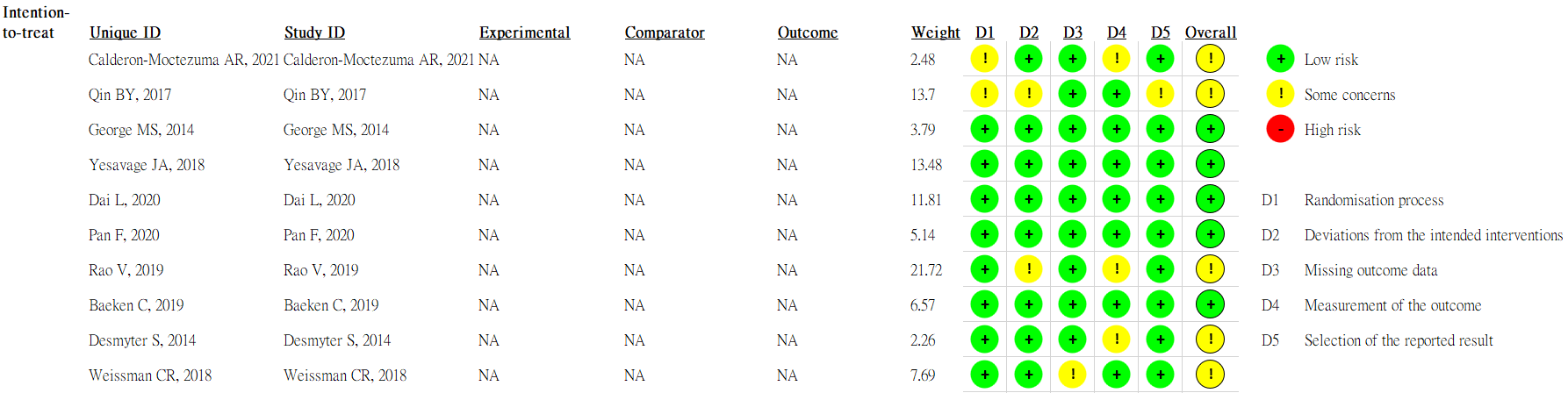


**Supplementary Table S6A:** Random-effects meta-regression analyses of potential moderators of the association of rTMS and reduction of suicide ideation

| **Clinical variables** | **Number of datasets** | **Slope; 95% CI** | ***P*-value** |
| --- | --- | --- | --- |
| Age | 10 | -0.05; -0.012 to 0.004 | 0.188 |
| **Female (%)** | **10** | **-0.994; -1.667 to -0.09** | **0.004*** |
| **Baseline BSI score** | **6** | **-0.031; -0.057 to -0.006** | **0.016*** |
| Baseline equivalent HAMD-17 score | 9 | -0.019; -0.076 to 0.037 | 0.501 |
| Treatment duration | 10 | 0.011; -0.004 to 0.026 | 0.142 |
| HAMD-17 score change | 8 | -0.033; -0.093 to 0.027 | 0.284 |
| Pulses per section | 11 | -0.00006; -0.00014 to 0.00002 | 0.133 |

* *p*<0.05

BSI: Beck scale for suicide ideation; CI: confidence interval; HAMD: Hamilton Depression Rating Scale; r-TMS: repetitive transcranial magnetic stimulation; MT: motor threshold

**Supplementary Table S6B:** Random-effects meta-regression analyses of potential moderators of the association of rTMS and reduction of depression severity

| **Clinical variables** | **Number of datasets** | **Slope; 95% CI** | ***P*-value** |
| --- | --- | --- | --- |
| Age | 9 | 0.003; -0.009 to 0.015 | 0.629 |
| **Female (%)** | **9** | **-1.226; -1.968 to -0.483** | **0.001*** |
| **Baseline equivalent HAMD-17 score** | **9** | **-0.109; -0.174 to 0.044** | **0.001*** |
| Treatment duration | 9 | -0.007; -0.028 to 0.014 | 0.507 |
| Pulses per section | 10 | 0.00001; -0.00008 to 0.00011 | 0.767 |

* *p*<0.05

CI: confidence interval; HAMD: Hamilton Depression Rating Scale; r-TMS: repetitive transcranial magnetic stimulation; MT: motor threshold

**Supplementary Table S7 -**Attrition and adverse events

| Author, year, study design | Attrition (N/baseline sample, %) | Adverse events (description, N) |
| --- | --- | --- |
| Calderon-Moctezuma AR, 2021, borderline personality disorder | Intervention group: 2/9=22.2%  Placebo group: 2/9=22.2% | Intervention group: General headache:2  Placebo group: General headache:1; Dizziness:1; Local discomfort in application area: 2 |
| Qin BY, 2017, depression | Intervention group: 5/85=5.9%  Placebo group: 2/100=2% | Intervention group: Dizziness, Nausea, Vomitting: 5  Placebo group: Nausea, Constipation, Headache, Dry mouth: 3 |
| George MS, 2014, PTSD or traumatic brain injury | Intervention group: 11/20=55%  Placebo group: 7/21=33.3% | Intervention group: Diplopia: 1, Brain contusion: 1, Back pain: 1, Dizziness: 1, Headache: 5, Erythema: 1  Placebo group: Eye pain: 1, Vision blurred: 1, Nausea: 2, Vomitting: 2, Myokymia: 1, Dizziness: 2, Headache: 4, Migraine: 1, Hypertension: 1 |
| Yesavage JA, 2018, TRD | Intervention group: 21/81=26%  Placebo group: 18/83=21.7% | Intervention group: Nasopharyngitis: 8, Depression: 8, Falls: 3, Headache: 15, Abnormal hearing test: 18, Suicide ideation: 3  Placebo group: Nasopharyngitis: 8, Depression: 3, Falls: 7, Headache: 16, Abnormal hearing test: 18, Suicide ideation: 4 |
| Dai L, 2020, elderly depression | Intervention group: 14/62=22.6%  Placebo group: 7/62=11.3% | Intervention group: Dizziness, Nausea, and Chest tightness: 5, Mild headache: 4  Placebo group: Nausea, Mouth dryness, Constipation, and Headache: 3 |
| Pan F et al, 2020, MDD | Intervention group: 0/21=0%  Placebo group: 0/21=0% | Intervention group: Mild headache: 4, sleepy: 3  Placebo group: |
| Rao V, 2019, MDD after TBI | Intervention group: 4/17=23.5%  Placebo group: 0/17=0% | Intervention group: Headache, Depression, Anxiety, Dizziness, Blurred vision, Fatigue, Puffy face, Eye twitching  Placebo group: Headache, Anxiety, Dizziness, Depression, Sleep problems, Face twitching |
| Baeken C, 2019, TRD | Intervention group: 0/21=0%  Placebo group: 0/24=0% | Local discomforts at the stimulation site during treatment or headache during or after the session were mentioned, but these complaints disappeared spontaneously after a couple of hours or a single intake of paracetamol |
| Desmyter S, 2014, TRD | Intervention group: 0/6=0%  Placebo group: 0/6=0% | Some patients mentioned some local discomfort at the stimulation site during treatment or headache during or after the session but these complaints disappeared spontaneously after a couple of hours or after a single intake of paracetamol. |
| Weissman CR, 2018, TRD | Intervention group: 14/48 + 11/80 = 25/128 = 19.5%  Placebo group: 5/20 + 5/41= 10/61 = 16.4% | Intervention group: Myocardial infarction: 1, Suicidality requiring hospitalization: 1, Insomnia: 1, Persistent scalp discomfort: 1, Recurrent headaches: 1, Anxiety: 4, Headache: 14, Pain:15  Placebo group: suicidality requiring hospitalization: 1, Headache: 14, Pain: 2 |

**Supplementary Figure S1 -**Forest plot of meta-analysis of improvement in suicide ideation scales in patients with suicide ideation receiving traditional repetitive transcranial magnetic stimulation therapy and those with control treatment

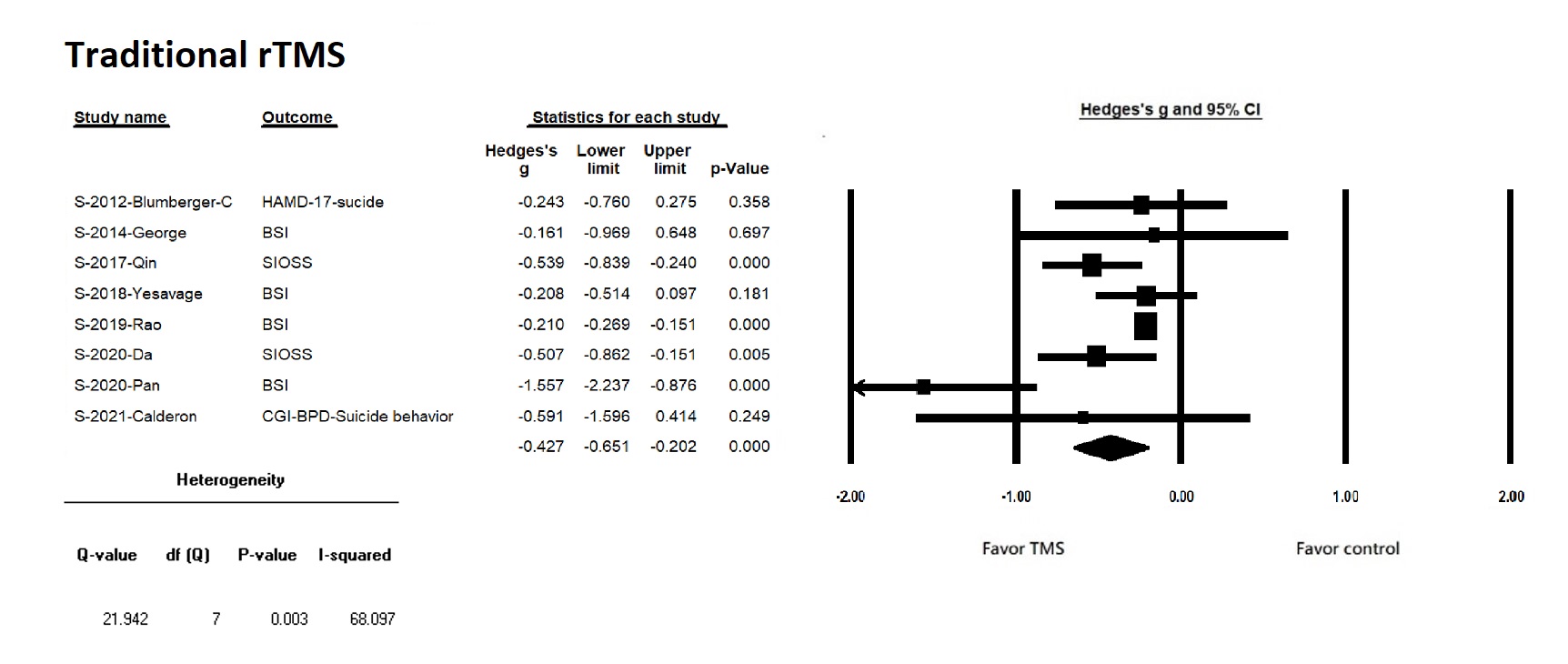


**Supplementary Figure S2 -**Forest plot of meta-analysis of improvement in suicide ideation scales in patients with suicide ideation receiving repetitive transcranial magnetic stimulation therapy target on left DLPFC and those with control treatment **
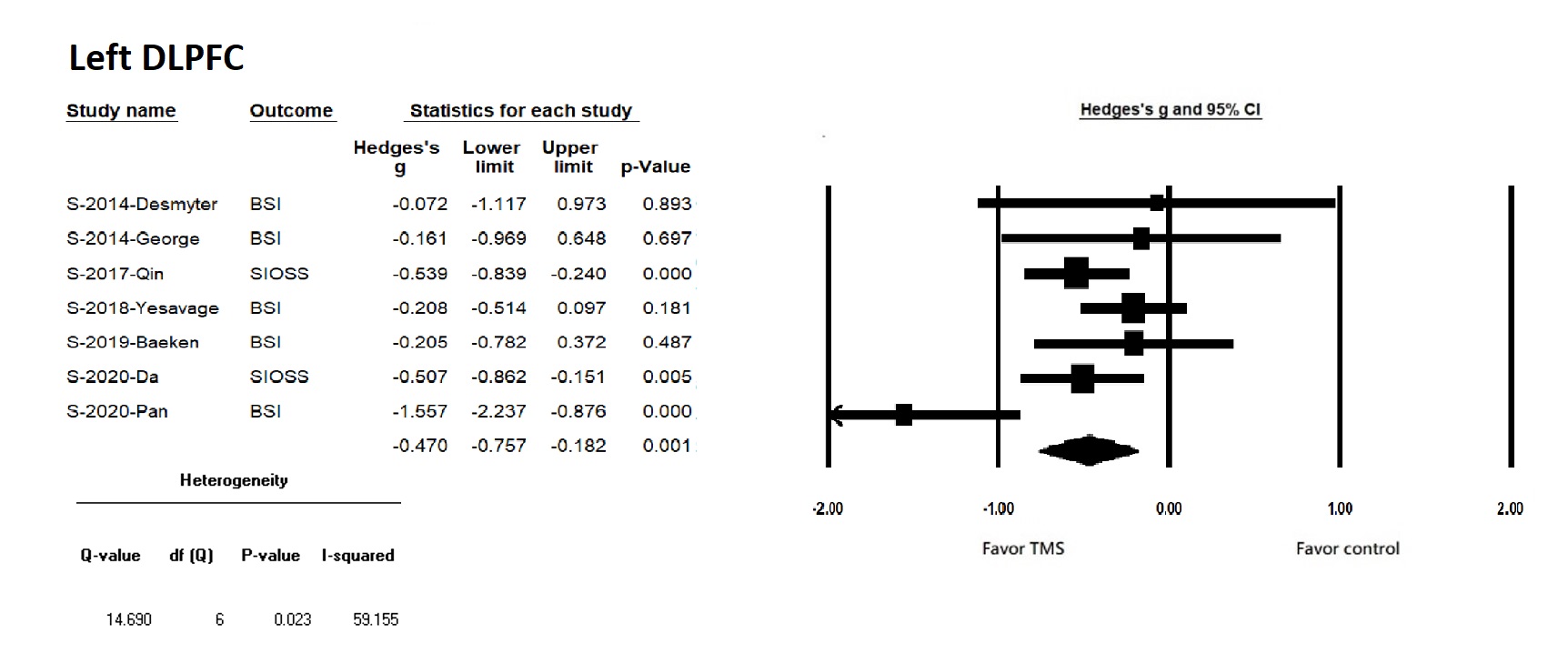
**
